# Research barriers in the Global South: Mexico

**DOI:** 10.7189/jogh.12.03032

**Published:** 2022-06-08

**Authors:** Alejandro Quiroga-Garza, Andrea N Garza-Cisneros, Rodrigo E Elizondo-Omaña, Jose F Vilchez-Cavazos, Roberto Montes de-Oca-Luna, Eliud Villarreal-Silva, Santos Guzman-Lopez, Jose G Gonzalez-Gonzalez

**Affiliations:** 1 Universidad Autonoma de Nuevo Leon, School of Medicine, Human Anatomy Department, Monterrey, Mexico; 2 Instituto Mexicano del Seguro Social, Delegación de Nuevo Leon, Monterrey, Mexico; 3 Universidad Autonoma de Nuevo Leon, University Hospital “Dr. Jose Eleuterio Gonzalez”, Traumatology and Orthopedic Surgery Departament, Monterrey, Mexico; 4 Universidad Autonoma de Nuevo Leon, School of Medicine, Histology Department, Monterrey, Mexico; 5 Ministry of Health of the State of Nuevo Leon, Monterrey, Mexico; 6 Universidad Autonoma de Nuevo Leon, University Hospital “Dr. Jose Eleuterio Gonzalez”, Neurosurgery Department, Monterrey, Mexico; 7 Universidad Autonoma de Nuevo Leon, University Hospital “Dr. Jose Eleuterio Gonzalez”, Endocrinology, Monterrey, Mexico; 8 Universidad Autonoma de Nuevo Leon, School of Medicine and University Hospital “Dr. Jose Eleuterio Gonzalez”, Research Vice-Dean’s Office, Monterrey, Mexico

The term Global South is used to denominate the regions outside Europe and North America (Latin America, Africa, Asia, and Oceania), economically, politically, or culturally marginalized [1,2]. One of the limitations of the Global South is the health inequities. There is a shortage of health care professionals, researchers, and economic resources in low- and middle-income countries (LMIC) [3-5]. The countries with the highest economic resources make up only 10% of the world population, yet these utilize at least 90% of the research funding designated annually for medical research. This implies that only 10% of the research funding is available to satisfy the needs for 90% of the rest of the world's population [6].

Latin American countries have made efforts to recruit students into research, but due to poor training, lack of resources, and deficient economic remuneration, many lose interest and few dedicate the time [7-9]. Studies in Columbia have reported that only 27% of professors have published a scientific paper, and only 5% in the prior year [8]. Others report that only 10.3% of research projects were published, of which most were in national journals, and only 11% of these were indexed in PubMed [10].

Mexico, one of the Global South countries with a population of 128 million, designates only 0.31% of its gross domestic product (GDP) to the science sector, compared to the 2.21% spent worldwide for research and development. High-income countries (HIC) such as the United States spend 2.8%, Germany 3.0%, and South Korea and Israel 4.9%. Despite this, the Mexican scientific community is the second major Latin American producer in terms of peer-reviewed academic publications, surpassed only by Brazil, which spends 1.28% of its GDP in research and development [11,12].

## BARRIERS IN MEXICO

Between 2011 and 2018, the number of articles produced by researchers in Mexico increased from 10 010 to 16 016, with an average annual growth rate of 7.1%. However, even though the absolute number of publications increased year after year, the percentage growth rate presented variations, and from the 22 research areas in which the Essential Science Indicators (ESI) scientific publications are classified, the Clinical Medicine and Psychology/Psychiatry areas had the lowest participation, with 0.50 and 0.49%, respectively [13].

It is important to improve the number of publications in Clinical Medicine in Mexico due to the plethora of pathologies in the country. Approximately 20% of the preventable deaths in Mexico are caused by diabetes and related metabolic diseases [14]. Between 2011 and 2020, the main causes of death in the population were heart disease, COVID-19, and Diabetes Mellitus [15]. In the population aged >50 years the most prevalent diseases were arterial hypertension (39.9%), Diabetes Mellitus (22.8%), and arthritis (11.2%) [15].

Type 2 Diabetes Mellitus (T2DM) is an alarming problem in Mexico and the world, but mostly in the countries that belong to the Global South due to environmental and genetic factors. Approximately 80% of the 415 million people with T2DM in the world live in LMIC, and 41.1 million of them live in Latin America. Mexico ranks second in Latin America with the highest prevalence of diabetes with 11.5 million cases, being surpassed only by Brazil, which has 14.3 million cases, and both are among the ten leading countries for the number of cases in the world [11,14].

The health system needs adjustments to prevent and treat the prevalent metabolic diseases in the Global South countries. Research to improve the populations' health and identify effective strategies is needed. However, important barriers can be identified in the health system. The three primary barriers to research innovation in Mexico are cultural, regulatory, and financial. The Mexican industry (health care corporations, funding agencies, biotechnology organizations, hospitals, and others) has not nurtured the culture of investing in research and innovation in the health care system. There is little participation from private corporations towards investment and a very unbalanced university system dominated by Social Sciences [16-18].

**Figure:**
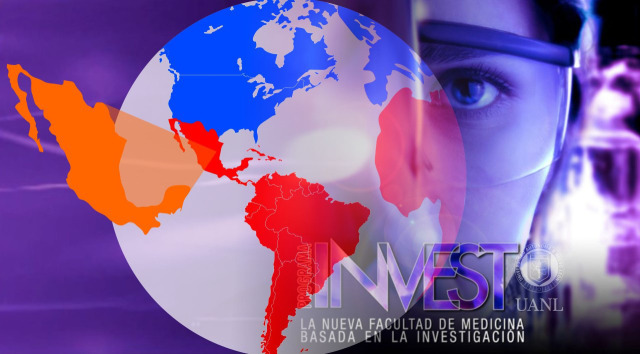
Photo: Mexico, a rising country of the research Global South. Source: Facultad de Medicina, Universidad Autonoma de Nuevo Leon, Monterrey, Mexico. Image Design & Editing by Armando Gibran Franco Salazar. Used with permission.

Since the 19^th^ century, the basic model of German teaching in universities has been based on a close union between teaching and research. In the 1950s, this teaching model changed in Mexico. With the creation of research centers and institutes, universities decreased the amount of research performed [16]. Many of the Mexican universities focused on teaching, with little to no formal training in research methods [19,20]. Few students have expressed interest in research, and fewer achieve a scientific publication as an author [21,22]. Without the dissemination of their research, the information and knowledge generated becomes stagnant and obsolete, contributing further to the dissatisfaction, burnout, and shortage of health care professionals and researchers [4,22,23].

Although certified schools promote research methodology and biostatistics education, the teachings are limited to the early years along with basic sciences. The lack of students’ experience leads to a low understanding and interest in the topic. Medical schools must improve these subjects, not only to encourage research, but to be able to understand and criticize published articles. This will lead to the formation of better physicians with the ability to integrate new evidence into their clinical decisions making [24-26].

A review of the curricula of all Mexican medical schools concluded that less than half have a medical statistics course [25]. Formal training programs are essential for the success of research development. Although efforts are being made in many teaching hospitals and institutes, there is still an important deficit [20,27].

Research is also stumped by the lack of respected time. Although Universities promote research among their educators, few assign a specific time for this activity. This is most evident in hospitals. If clinicians desire to work on research projects or publish, they must use their time outside work or rush their work to create time, as their clinical responsibilities and activities remain the same as those who do not participate in research.

## GOVERNMENT ROLE IN SCIENTIFIC OUTPUT

The National Council of Science and Technology (CONACyT) was created in 1970 in Mexico. This government agency manages national scientific policy. Its main functions include:

- financial support of research centers and universities that meet the evaluation criteria of international excellence.
- creating a scholarship system that promotes the training of new researchers in public universities and regional technological institutes and a scholarship system for researchers linked to international performance parameters.
- support of projects that investigate national problems.

From 1396 researchers in 1984, it currently has over 30 000 researchers as members of the National System of Researchers (SNI [*Sistema Nacional de Investigadores*]). Currently, to become a member, an individual must have a PhD and evidence of a trajectory of research, capacity to publish, and creation of human resources in professional activities. However, these include mathematics, earth sciences, biology, chemistry, medicine, health sciences, social and human sciences, biotechnologies, and engineering. Medicine and health sciences rank among the lower categories with regard to the number of researchers with 11.5% [8,16,28].

In 2019, the financial funding for the research centers supported by CONACyT decreased by 50%, and the overall budget was cut by 12% [12]. These budget cuts hamper the education system by affecting undergraduate schooling, and the recruitment of early-career researchers back to Mexico [12,22,29].

To promote innovation, it is necessary to identify the variables associated with positive research attitudes and productivity among medical students. Currently, students perceive a lack of time and the lack of mentors as their primary limitations. Mentors and peers already involved in research are the primary sources of motivation for student recruitment, with positive results in the early years of their careers [22,30,31]. Studies have shown most physicians who are also researchers began participating in research during medical school as collaborating students [32-34].

Educational programs should also be updated in Mexico. Strategies such as teaching based on the review of published articles, use of clinical reasoning, case-based teaching, and flipped-classroom techniques could aid in increasing the students’ understanding of evidence-based medicine [35-37]. Research methodology should be re-visited in clinical years. All Universities should be encouraged to incorporate training courses and perform clinical or epidemiologic research under the surveillance of an ethics and research committee.

Government should also facilitate the collaboration between Universities and the private sector. There is a lack of research funding in Mexico. A higher percentage of the GDP should be invested in CONACyT and the SNI. Although the current government has promised an increase in the Expenditure on Research and Experimental Development to at least 1%, the goal has not been reached [27].

## CREATING RESEARCHERS – UANL STRATEGIES

The key to success has been the prioritization of research as an independent branch, with the absence of politics. The Universidad Autónoma de Nuevo León (UANL) medical school had the clear objective of implementing change to become a research-oriented institution. In 2005, it created the research vice dean’s office. This department aided educators and clinicians in conducting high-quality research under the supervision of the corresponding review boards. It also established the resources to guide researchers in improving their work and training new generations.

Courses were made available periodically for free (ie, use of search engines, study design, methodology, statistical analysis, manuscript editing, submitting articles, etc.). Expert advice and consults could be scheduled. English translation and editing services were free of charge. Availability of collaborating with engineering for innovation design, along with legal orientation for patent registration, and developing consent forms. It incorporated a clear link with the private industry and pharmaceutical companies for collaboration, which also created economic resources for funding. Specialized administrative personnel was trained to identify research opportunities and federal grant announcements with the UANL, as well as guide professors on how to apply to the SNI. In its first 4 years, the number of researchers in the school increased from 30 to 80, a 266% increase. Currently, over 65% of the 329 educators/professors are members of the system and actively work in research [38].

Other well-established research groups within the University had promoted student involvement with established authorship and collaborative mentoring to create young researchers [39]. The anatomy research group (GIA [*Grupo de Investigación en Anatomía*]) established in 2003, has a well-structured program that mentors undergraduate medical students in educational research and anatomical research with clinical orientation, with authorship in their scientific production. In the last 5 years, the group has produced over 50 publications in indexed journals, and many more in other national and international peer-reviewed journals, as well as student participation with oral and poster presentations in meetings. With this in mind, the Vice Dean’s office launched the “BP Invest” program in 2012, where it gathers its best students with its best professors to promote research and innovation. Through a rigorous program, these students will learn and put into practice their skills to aid other students and researchers in their work and become published authors. To promote student involvement, it designed the “research trinomial [*trinomio perfecto*)” in which all research projects must involve at least one professor, a post-graduate student, and a medical student. This encourages the collaboration and inclusion of research at all levels of professional training. It also implemented a contact database (Affinity Invest) for researchers and students with similar interests to communicate and collaborate. Although student involvement continues to be low (2.5%) due to a very large student volume (>7500 students enrolled in the 6-year program), the majority of those involved (60%) have established authorship in a published paper [22,39].

The link between research and the post-graduate program vice deans’ offices has also driven the publication of nearly all theses in indexed journals. Many students and educators have been given scholarships and grants to complete research training abroad in centers of evidence-based medical research. Due to the constant training of its personnel, in 2018, the KER Unit Mexico was inaugurated in the UANL, creating a direct link with Mayo Clinic in Rochester, Minnesota, USA. This Unit trains students and professors in systematic reviews and meta-analyses, creating the opportunity to collaborate internationally (Mayo Clinic, McMaster University, Yale University, Miami University, Cracovia University, etc.) with over 60 papers published with the aid of the unit, in the last 3 years [40].

The commitment and strategies implemented by the UANL have made it an important source of research in Mexico. Over 600 papers are published annually in indexed journals by the medical school. Over 25 million pesos have been granted for research [38].

## FUTURE PERSPECTIVES

In the future, the UANL medical school will have exponential growth of researchers secondary to the strategies of involving medical students. It projects an increase in its links with the private sector, and it will increase its scientific productivity originated from the talent, capability, dedication, and originality of its professors. There is no doubt the future is in the investment of student involvement. Formal groups and training increase the probability of publishing by 180% [7]. Prospective studies will be needed to evidence the scientific production of clinicians who are currently part of well-established research groups.

The Mexican government needs to increase investment in health research, not only due to the high prevalence of metabolic diseases in the country but also to combat emerging ones, such as the COVID-19 pandemic. The frequent change of political parties in the executive power branch has also shifted the availability of resources.

In 2020, CONACyT had a budget of 25 658.8 million Mexican pesos (MXP) (approximately US$1364.8 million), of which 24.1% were designated for public research centers. A total of 5389.2 million MXP ( = US$286.7 million) was assigned to the SNI program, which supported 32 389 scientists and technologists who were members of the program. Also, a budget of 1345.1 million MXP ( = US$71.5 million) was allocated to the CONACyT Chairs, supporting 1511 positions for professors. With the SARs-CoV-2 (COVID-19) pandemic outbreak, it designated 22 million MXP ( = US$1.2 million) in grants for COVID-19 research [41,42].

Global South countries must strive to increase the quality of their research, to demonstrate the value of their work. Although an increase in GDP investment by the government, and higher collaboration with the private sector may aid in creating resources, the key to success rests on the current administrative personnel, researchers, and mentors. They must be creative and committed to generating research strategies early in the training of professionals. They must create research activities focused on the main causes of death in their countries; generating knowledge and applying it to its inhabitants. Researchers should publicly acknowledge and support young researchers (students/residents/fellows) with authorship, to inspire others. Global South countries must not interrupt the interest of training young researchers, and focus on the interest for the growth of research. By focusing on these challenges and solving these limitations, Global South countries will be able to improve public health and benefit millions of patients.
